# Traumatic injuries to the renal blood vessels and in-hospital renal complications in patients with penetrating or blunt trauma

**DOI:** 10.3389/fsurg.2023.1134945

**Published:** 2023-05-31

**Authors:** Nasser A. N. Alzerwi

**Affiliations:** Department of Surgery, College of Medicine, Majmaah University, Ministry of Education, Al-Majmaah City, Riyadh Region, Saudi Arabia

**Keywords:** renal blood vessels, renal dysfunction, NTDB, cardiac arrest, trauma

## Abstract

**Background:**

Traumatic injuries to renal blood vessels (IRBV) can have significant consequences for patients, impacting their mortality, morbidity, and quality of life.

**Objective:**

This study aimed to compare trauma types and injury characteristics, vital signs, and outcomes in patients with and without IRBV (nIRBV) and examine whether IRBV and pre-existing renal dysfunction affected the likelihood of in-hospital renal complications (iHRC).

**Materials and Methods:**

After identifying penetrating and blunt trauma victims with IRBV in the National Trauma Data Bank, patient demographics, injury-related variables, treatment outcomes, and deaths under care were analyzed and compared.

**Results:**

Of the 994,184 trauma victims, 610 (0.6%) experienced IRBV. Victims in the IRBV group (IRBVG) had a significantly higher frequency of penetrating injuries (19.5% vs. 9.2%, *P* < 0.001) and higher injury severity score (ISS ≥25, 61.5% vs. 6.7%). Most injuries in both groups were unintentional, although a higher frequency of assault was noted in the IRBVG. The incidence of iHRC was higher in the IRBVG (6.6%) than in the nIRBVG (0.4%; *P* < 0.001). The IRBV {OR = 3.5 [95% CI = (2.4–5.0)]}, preexisting renal disorders {OR = 2.5 [95% CI = (2.1–2.9)]}, and in-hospital cardiac arrest {OR = 8.6 [95% CI = (7.7–9.5)]} were found to be among the factors associated with a higher risk of iHRC.

**Conclusions:**

IRBV and pre-existing renal disorders considerably increased the risk of developing iHRC. Due to the long- and short-term consequences of associated cardiovascular, renal, and hemodynamic complications, victims of IRBV require specialized renal management and close monitoring.

## Introduction

Despite their relatively safe retroperitoneal positioning, the kidneys are the most probable genitourinary organ to be damaged by trauma (1–3). The vasa recta descend into the medullary sections of the kidney and is part of a highly vascularized network that connects cortical glomerular structures (4–8). Studies indicate that renal trauma may induce irreversible damage to renal vascular integrity, resulting in both short- and long-term repercussions such as high blood pressure, kidney damage, and kidney failure (9–14).

It has been reported that specific preexisting renal injuries (such as cysts or hydronephrosis) (15) may amplify the impact of trauma, and traumatic irbvs and preexisting renal complications may increase the predisposition of trauma victims to in-hospital renal complications (16). Unfortunately, although there are few case reports and retrospective investigations of renal trauma, the incidence and features of IRBV are primarily absent from the literature. Therefore, it is difficult to provide suggestions for optimal management of IRBV. Using a nationwide database to identify trauma characteristics and factors associated with hospital-acquired renal issues following IRBV may be vital for gaining greater insight into this understudied field (17).

Recently, Owattanapanich et al. Investigated isolated blunt renal artery injury using the National Trauma Data Bank (NTDB) and identified several factors associated with managing blunt renal artery injury (BRAI) (17). Although Owattanapanich et al. Provided a critical perspective on the management of BRAI, they did not include all renal vascular injuries and penetrating trauma. Similarly, in a seminal study, Sangthong et al. Explored BRAI by conducting an extensive analysis of 517 patients from NTDB, revealing considerably longer hospital and ICU stays in the case of surgical revascularization (7). Both these studies highlighted the need for specific care for renal trauma patients with renal arterial injuries. However, both studies focused only on blunt trauma and the renal arteries.

Studies on IRBV focusing on in-hospital and pre-existing renal complications are lacking in the literature. Considering the high prevalence of in-hospital complications in trauma victims (18) and the different trauma mechanisms and pathogenesis associated with different trauma types (19, 20), such as penetration, it is important to examine IRBV in the context of different trauma types, mechanisms, and intent. Such an analysis can provide vital information for managing and understanding in-hospital complications. There is a pressing need to investigate IRBV and pre-existing renal issues due to the long-term ramifications of in-hospital renal problems after traumatic injuries (14, 21).

This study aimed to investigate the impact of irbvs on the likelihood of in-hospital renal complications (ihrc) in trauma patients. In addition, this study aimed to determine whether patients with pre-existing renal complications are more susceptible to irbvs and ihrc after trauma. This study provides novel insights into the prevalence of traumatic IRBV, patient demographics, injury-associated factors, ihrc, and mortality rates. Furthermore, this study identified factors associated with the risk of ihrc, such as IRBV, pre-existing renal disorders, and in-hospital cardiac arrest. The significance of this study lies in its potential to improve the management and care of trauma patients with IRBV and preexisting renal complications. By identifying the risk factors for ihrc, health care providers can better monitor and manage these patients, leading to improved outcomes and quality of life. The findings of this study also highlight the need for specialized renal management for patients with IRBV due to the potential long- and short-term cardiovascular, renal, and hemodynamic complications. Ultimately, this study sheds light on an understudied area of trauma care, providing crucial insights into the management of patients with traumatic IRBV and preexisting renal complications.

## Materials and methods

### Study population

We searched the 2017 NTDB for trauma victims. Patients with IRBV injury codes defined by the International Classification of Diseases, Tenth Revision, Clinical Modification (ICD-10-CM) were included in the IRBV group (IRBVG), whereas all other patients were included in the nirbv group (nirbvg). The investigation included patients who experienced penetrating and blunt trauma and any mechanism of injury. The demographic data included patient age, sex, and race. Injury mechanism and intent, Associated Injury (AIS) for body area injuries, injury severity score (ISS), shock, and total Glasgow Coma Scale (GCS) scores were included in the injury profile.

### Outcomes

The main outcome was renal complications at the hospital. The secondary outcomes were in-hospital mortality, significant complications (pulmonary embolism (PE), deep vein thrombosis (DVT), myocardial infarction (MI), pneumonia, sepsis, and stroke), hospital length of stay (LOS), and intensive care unit length of stay (ICU LOS). Patients with missing data were excluded from the analysis.

### Statistical analysis

All patient characteristics in the total injury population stratified into the IRBV and nirbv groups were presented using descriptive statistics. Categorical data are reported as frequencies and percentages, and medians and interquartile ranges (IQR) are used to report continuous data. The ihrc and secondary outcomes were assessed for the IRBVG and nirbvg. Descriptive analysis was performed and differences were compared using the Kruskal–Wallis test. Multivariable analysis was performed to identify independent variables. The following variables were included: age ≥65 years, GCS score, ISS, preexisting renal complications, and in-hospital cardiac arrest. Statistical significance was set at *P* < 0.05. Statistical analyses were performed using Stata (version 12.0; Stata Corp, College Station, TX, USA).

## Results

### Prevalence, demographics, injury characteristics, and vital signs

Of 994,184 trauma victims, 603 (0.06%) experienced IRBV (Table 1). The patients in the IRBVG were younger than those in the nIRBVG (median age (IQR):32.0 (23.0, 47.0) vs. 49.0 (26.0, 69.0), *P* < 0.001). Notably, there were no significant differences in the frequency of pre-existing renal conditions between the two groups (*P* = 0.158). Victims in the IRBVG had a significantly higher prevalence of penetrating injuries (19.8% vs. 9.2%, *P* < 0.001). Most injuries in both groups were unintentional, although a higher frequency of assault was noted in the IRBVG (18.4% vs. 9.7%, *P* < 0.001). The trauma mechanism also differed considerably between the groups (*P* < 0.001), with significantly higher contributions from the firearm (17.3% vs. 4.5%) and motor vehicle traffic (MVT) (57.9% vs. 26.5%) mechanisms in the IRBVG.

**Table 1:** Comparison of demographics, trauma intent, and mechanism.

	nIRBV (*N* = 9,93,581)	IRBV (*N* = 603)	*P*-value
Age, Years, Median (Q1, Q3)	49.0 (26.0, 69.0)	32.0 (23.0, 47.0)	<0.001
Age group			<0.001
<16	98,040 (10.0%)	25 (4.2%)	
16–20	48,856 (5.0%)	68 (11.4%)	
21–44	2,68,613 (27.3%)	324 (54.5%)	
45–64	2,24,196 (22.8%)	118 (19.9%)	
65+	3,44,062 (35.0%)	59 (9.9%)	
Sex (Men)	5,97,351 (60.1%)	434 (72.0%)	<0.001
Injury type			<0.001
Blunt	8,68,027 (87.8%)	481 (79.9%)	
Penetrating	90,883 (9.2%)	119 (19.8%)	
Others	29,827 (3.0%)	2 (0.3%)	
Mechanism			<0.001
MVT	2,61,024 (26.5%)	342 (57.9%)	
Firearms	43,927 (4.5%)	102 (17.3%)	
Fall	4,58,460 (46.6%)	65 (11.0%)	
Cut/Peirce	48,936 (4.9%)	17 (2.9%)	
Others	1,72,494 (17.5%)	65 (11.0%)	
Intent			<0.001
Unintentional	8,75,487 (88.1%)	474 (78.6%)	
Self-inflicted	13,888 (1.4%)	13 (2.2%)	
Assault	96,431 (9.7%)	111 (18.4%)	
Others	7,630 (0.8%)	5 (0.8%)	
Preexisting renal conditions	15,261 (1.5%)	5 (0.8%)	0.158

nIRBV, victims without injury of renal blood vessels; IRBV, victims with injury of renal blood vessels; MVT, Motor Vehicle Trauma.

The time to emergency medical services (EMS) response did not vary significantly between the groups (*P* = 0.444, Table 2). A helicopter ambulance was used in 19.4% of the IRBV patients, compared to 7.5% of the cases in the nIRBVG (*P* < 0.001). In the IRBVG, 6.1% of the victims had no signs of life, in contrast to a significantly lower proportion (0.8%) in the nIRBVG (*P* < 0.001). Systolic blood pressure (SBP) and GCS score were significantly lower in the IRBVG than in the nIRBVG, but ISS was significantly higher in the IRBVG than in the nIRBVG (both *P* < 0.001).

**Table 2:** Comparison of emergency response time, vital signs, and renal injuries.

Median (Q1, Q3) or %	nIRBV (*N* = 9,93,581)	IRBV (*N* = 603)	*P*-value
Time to EMS Response (mins)	8.0 (5.0, 14.0)	8.0 (5.0, 14.0)	0.444
SBP	136.0 (120.0, 154.0)	116.5 (96.0, 138.0)	<0.001
Pulse rate	87.0 (74.0, 101.0)	98.0 (80.0, 116.0)	<0.001
Respiratory rate	18.0 (16.0, 20.0)	20.0 (16.0, 24.0)	<0.001
Total GCS	15.0 (15.0, 15.0)	15.0 (7.0, 15.0)	<0.001
ISS	8.0 (4.0, 10.0)	27.0 (19.0, 38.0)	<0.001
SBP < 90	28,369 (2.9%)	114 (18.9%)	<0.001
GCS ≤ 8	55,468 (5.6%)	159 (26.4%)	<0.001
ISS Groups			<0.001
1–8	5,00,547 (50.4%)	0 (0.0%)	
9–15	3,31,775 (33.4%)	81 (13.4%)	
16–24	95,111 (9.6%)	149 (24.7%)	
25+	66,148 (6.7%)	373 (61.9%)	
Transport mode			<0.001
Ground Ambulance	7,44,054 (75.4%)	440 (73.5%)	
Helicopter Ambulance	74,333 (7.5%)	116 (19.4%)	
Fixed-wing Ambulance	4,088 (0.4%)	7 (1.2%)	
Private/Public Vehicle/Walk-in	1,55,997 (15.8%)	28 (4.7%)	
Police	3,331 (0.3%)	3 (0.5%)	
Other	4,461 (0.5%)	5 (0.8%)	
Interfacility transfer	7,37,051 (74.2%)	490 (81.3%)	<0.001
Emergency department disposition			<0.001
Floor	4,14,675 (42.9%)	41 (6.9%)	
Intensive care unit	1,83,828 (19.0%)	228 (38.3%)	
Operating room	1,08,041 (11.2%)	247 (41.5%)	
Home	91,638 (9.5%)	0 (0.0%)	
Dead	10,144 (1.1%)	40 (6.7%)	
Other	1,57,664 (16.3%)	39 (6.6%)	
Arrived with no signs of life	7,736 (0.8%)	37 (6.1%)	<0.001
High-grade kidney injuries	1,725 (0.2%)	81 (13.4%)	<0.001
IRBV type			.
Laceration of renal artery	0 (.%)	241 (40.0%)	
Laceration of right renal vein	0 (.%)	93 (15.4%)	
Others	0 (.%)	269 (44.6%)	
Associated Injury regions (AIS ≥ 3)			
1. Head/Neck	1,48,495 (14.9%)	49 (8.1%)	<0.001
2. Face	1,595 (0.2%)	1 (0.2%)	0.974
3. Chest	1,28,140 (12.9%)	324 (53.7%)	<0.001
4. Abdomen	42,159 (4.2%)	603 (100.0%)	<0.001
5. Extremities	1,70,580 (17.2%)	156 (25.9%)	<0.001
6. External	3,883 (0.4%)	0 (0.0%)	0.124

nIRBV, victims without injury of renal blood vessels; IRBV, victims with injury of renal blood vessels; SBP, Systolic Blood Pressure; GCS, Glasgow Coma Scale; ISS, Injury Severity Score; AIS, Abbreviated Injury Scale.

Concurrent injuries in different body locations differed significantly between the IRBV and nIRBVGs; 53.7% of patients with IRBV had an associated injury in the thoracic region (AIS ≥ 3) compared with 12.9% of patients in the nIRBVG (*P* < 0.001, Table 2). All patients in the IRBVG had abdominal trauma(AIS ≥ 3) compared with 4.2% in the nIRBVG (*P* < 0.001). Moreover, 25.9% of patients in the IRBVG had associated extremity injuries compared to 17.2% in the nIRBVG (*P* < 0.001). The percentage of high-grade renal injuries was 13.4% in the IRBVG and 0.2% in the nIRBVG (*P* < 0.001). Among IRBVs, 40.0% involved laceration of the renal artery, 15.4% involved laceration of the right renal vein, and 44.6% involved other IRBV types.

### Primary outcome

The IRBVG had a greater prevalence of iHRC (6.6%) than the nIRBVG (0.4%; *P* < 0.001; Table 3).

**Table 3:** Comparison of the outcome.

Median (Q1, Q3) or %	nIRBV (*N* = 9,93,581)	IRBV (*N* = 603)	*P*-value
Total ICU LOS	3.0 (2.0, 5.0)	5.0 (3.0, 10.0)	<0.001
Length of stay (days)	3.0 (2.0, 6.0)	9.0 (3.0, 17.0)	<0.001
Hospital disposition			<0.001
Home	4,88,345 (57.6%)	225 (40.8%)	
Facility	1,28,339 (15.1%)	35 (6.4%)	
Dead	26,002 (3.1%)	100 (18.1%)	
Other	2,05,836 (24.3%)	191 (34.7%)	
Hospital Mortality	26,002 (2.6%)	100 (16.6%)	<0.001
Died Under Care	36,146 (3.6%)	140 (23.2%)	
In hospital complications			
Kidney	4,355 (0.4%)	40 (6.6%)	<0.001
Cardiac arrest	6,017 (0.6%)	50 (8.3%)	<0.001
Sepsis	2,485 (0.3%)	10 (1.7%)	<0.001
Stroke/CVA	2,006 (0.2%)	7 (1.2%)	<0.001
Pneumonia	4,013 (0.4%)	14 (2.3%)	<0.001
MI	1,444 (0.1%)	2 (0.3%)	0.230
Pneumonia	2,609 (0.3%)	13 (2.2%)	<0.001

nIRBV, victims without injury of renal blood vessels; IRBV, victims with injury of renal blood vessels; Total ICU LOS, Total Intensive Care Unit Length of Stay; LOS, Length of Stay; Stroke/CVA, Stroke or cerebrovascular accident during hospitalization; MI, Myocardial infarction during hospitalization.

### Secondary outcomes

Overall, the proportion of patients who died under care (DUC) (22) was 23.2% (Table 3) in the IRBVG, which was higher than that in the nIRBVG (*P* < 0.001). Median LOS in the hospital was 9.0 (3.0, 17.0) days in the IRBVG and 3.0 (2.0, 6.0) days in the nIRBVG (*P* < 0.001). Median LOS in the ICU was 5.0 (3.0, 10.0) days in the IRBVG and 3.0 (2.0, 5.0) days in the nIRBVG (*P* < 0.001). Overall, 29.0% of patients with IRBVG experienced in-hospital complications. In contrast, 3.5% of complications were found in the nIRBVG. All complications were more frequent in the IRBVG than in the nIRBVG (Table 3). In the IRBVG, the frequency of cardiac arrest was 8.3% compared to 0.6% in the nIRBVG (*P* < 0.001).

### Blunt vs. penetrating trauma

**Figure 1:**
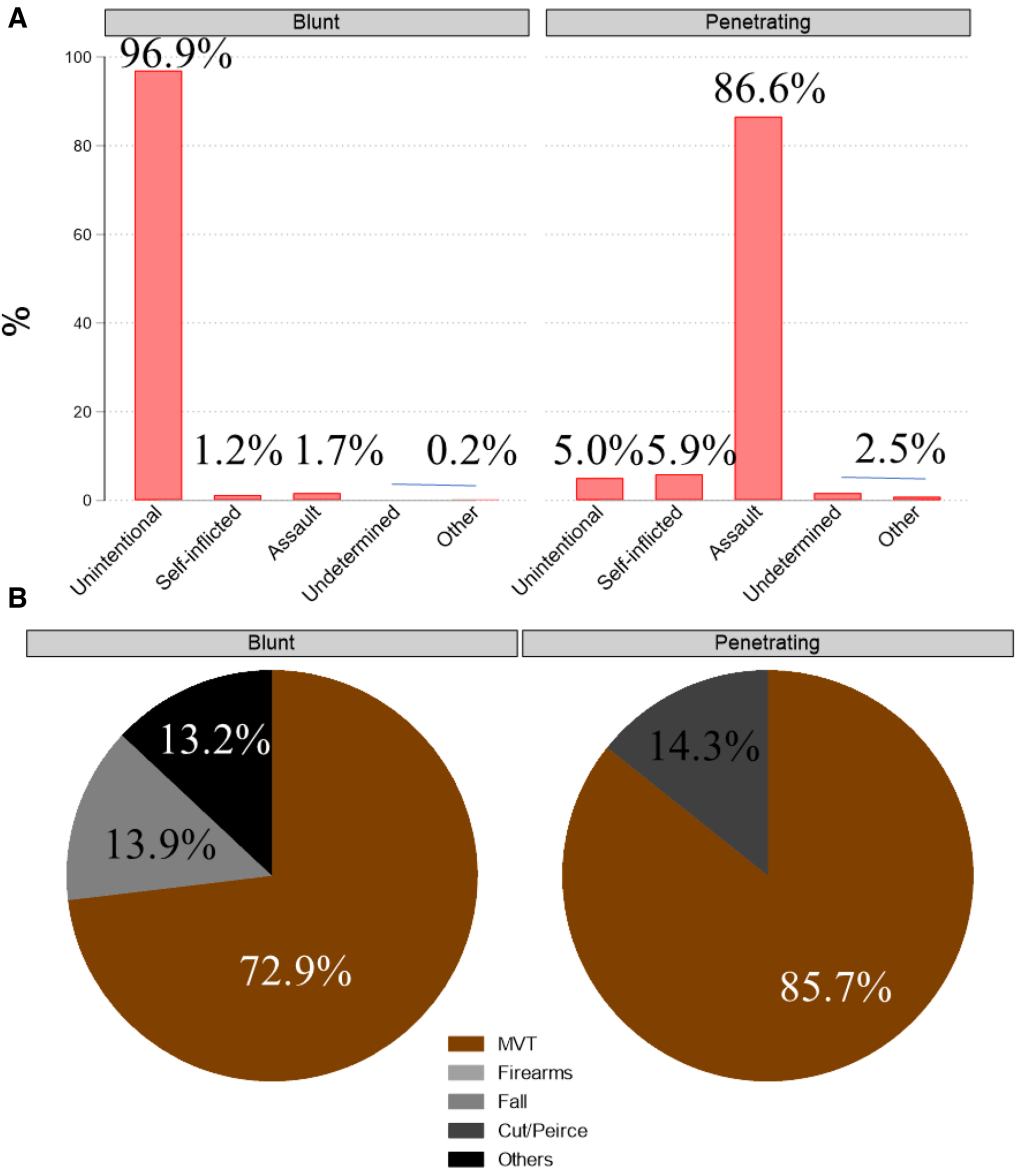
Comparison of trauma types in victims with injury of renal blood vessels after penetrating or blunt trauma (**A**) intent (**B**) mechanism.

Figure 1 shows the intent and mechanism of injury in patients with IRBV. Penetrating injuries were predominately caused by firearms (85.7%), whereas MVT was the primary cause of blunt trauma (72.9%). Moreover, most injuries in the case of blunt trauma were unintentional (96.9%), whereas most injuries in the case of penetrating trauma were due to assault (86.6%). The DUC was 41.2% for penetrating trauma and 18.5% for blunt trauma (*P* < 0.001). In-hospital renal complications did not differ between blunt trauma (6.1%) and penetrating trauma (8.4%) (*P* = 0.627).

### Risk factors for iHRC

Multivariable logistic regression also identified several factors associated with iHRC, which are presented in Table 4. Pre-existing renal conditions {OR =  2.4 [95% CI = (2.1–2.8)]}, IRBV {OR = 3.0 [95% CI = (2.1–4.3)]}, and high-grade renal injury {OR =  2.2 [95% CI = (1.7–2.9)]} were independently associated with a higher risk of iHRC. Age ≥65 years, ISS, GCS score, associated injury in the chest region, pre-existing renal complications, and in-hospital cardiac arrest were also significantly associated with the risk of in-hospital renal complications (area under the receiver operating characteristic curve = 0.7967).

**Table 4:** Logistic regression of in-hospital kidney complications in trauma victims.

Variables	Odds ratio	95% Confidence interval	*P*-value
Age ≥65	2.151	2.017–2.293	<0.01
Sex (women)	0.628	0.587–0.672	<0.01
SBP <90	1.151	1.028–1.289	0.015
GCS <9	1.355	1.228–1.494	<0.01
ISS	1.039	1.037–1.042	<0.01
Chest injury (AIS >3)	1.926	1.79–2.072	<0.001
Preexisting renal conditions	2.431	2.089–2.83	<0.01
IRBV	3.033	2.098–4.384	<0.01
High-grade renal injury	2.248	1.714–2.948	<0.01
In-hospital cardiac arrest	8.773	7.912–9.727	<0.01

OR, Odds Ratio; CI, Confidence Interval; SBP, Systolic Blood Pressure; GCS, Glasgow Coma Scale; ISS, Injury Severity Score; AIS, Abbreviated Injury Scale; IRBV, Injury of Renal Blood Vessel.

## Discussion

Renal trauma affects up to 3.25% of patients with trauma, with blunt trauma being the most common cause (2, 23). As the kidney is highly vascularized, IRBV may result in substantial short- and long-term repercussions. It has been stated that specific preexisting renal injuries (such as cysts or hydronephrosis) (15) may amplify the trauma impact and traumatic IRBVs; moreover, preexisting renal complications may enhance the predisposition of trauma victims to in-hospital renal complications (8, 16). Unfortunately, although there are few case reports and retrospective studies on renal trauma, the prevalence and characteristics of IRBV are largely missing from the literature.

This study investigated several trauma-related factors in renal blood vessel injuries. Most importantly, we sought to determine whether pre-existing renal complications or IRBV caused by trauma increase the likelihood of in-hospital renal complications. By including both blunt and penetrating traumas, we delineated the differences in trauma mechanisms, in-hospital complications, and in-hospital mortality. Our findings showed that IRBV and preexisting renal complications considerably increased the risk of in-hospital renal complications. However, pre-existing renal complications (i.e., renal diseases before trauma) did not vary significantly between IRBV and nIRBVGs.

It has been postulated that the pattern of the mechanism causing renal trauma may differ depending on the demographic characteristics (24, 25). In our study, blunt trauma accounted for 79.9% of IRBV cases; total MVT-related incidents accounted for more than 57.9% of all injuries in the current study, followed by gunshot injuries (17.3%) and falls (11%), which is in line with previous studies (24–26). In a meta-analysis, patients who experienced blunt trauma accounted for 80.5% of the cases, whereas penetrating mechanisms accounted for 19.5% (27). Another systematic review found that MVT (63%) was the leading cause of renal trauma in adults, followed by fall (43%) (28). The inclusion of penetrating trauma in our analysis might explain the disparity between this review and our findings. Previous findings were well validated when our population was categorized as having blunt or penetrating trauma. In blunt trauma, we found that MVT was the major mechanism, accounting for 73% of the cases, followed by fall injuries observed in approximately 14% of the cases. According to Georgitis et al., MVT causes the most occurrences of blunt kidney trauma, with arterial thrombosis being the most prevalent finding (29).

Similarly, Coccolini et al. stated that the most prevalent mechanism of renal damage is blunt trauma (90% of cases), which is commonly accompanied by high-velocity deceleration, whereas penetrating trauma is more severe and unpredictable than blunt trauma and occurring in 1.4%–3.3% (24). In our analysis, firearms accounted for approximately 85% of IRBVs in penetrating trauma, with stab wounds (cut/pierce) accounting for the remaining 15%. In penetrating trauma, the most prevalent intent was assault, accounting for 86% of cases. However, in blunt trauma, the intent was unintentional in most cases. In terms of the average ISS, Comma scale, and hemodynamic status, blunt and penetrating trauma victims with IRBV did not differ significantly. However, just 3.4% of penetrating trauma patients were above the age of 65; in contrast, 11.5% of blunt trauma patients were above the age of 65. These discrepancies may be attributed to the higher frequency of assault in cases of penetrating trauma, which is common in younger patients (27, 30).

In general, renal trauma mostly affects males, accounting for 72–93 percent of all victims, and is more prevalent among the young, in the age range of 31–38 years (1). Our findings support the notion that men outnumber women among IRBV patients and that more than half of the victims were between the ages of 21 and 44 years, which is also consistent with earlier studies (31). Notably, we found that age >65 years significantly increased the risk of in-hospital renal complications, even after adjusting for preexisting renal diseases and other factors. As kidney issues are more common in individuals 65 years and older, these data highlight the need to provide specialized renal treatment and close monitoring for patients with IRBV in the senior population (32, 33).

The incidence of iHRC was significantly higher in patients with IRBV (6.6%) than in those without IRBV (0.4%). According to Starnes et al., patients who underwent renal exploration, even those with mild or severe kidney injury, had more than double the local complication risk of patients who did not (34). Our findings support these statements in the setting of renal trauma, as multivariable logistic modeling demonstrated that IRBV might increase the likelihood of in-hospital clinical problems by a factor of two. Such patients should be considered for managed care and post-discharge monitoring in particular. In our study, the incidence of DUC in the IRBVG was 23.2%. Although epidemiological studies focusing exclusively on IRBV are not available, patients with renal trauma have reported a 30-day in-hospital mortality rate of 17% (25).

Our results confirmed that the trauma mechanism and intent differ significantly in blunt and penetrating trauma. However, the pathophysiology of blunt renal trauma remains unclear. Nevertheless, deceleration and acceleration forces are anticipated to contribute significantly to injury by driving the kidney to collide with surrounding organs (35). Penetrating trauma, on the other hand, is categorized based on projectile velocity: high-velocity weapons inflict more severe damage because the bullets transfer enormous amounts of energy to the organs. Far from the path of the bullet, cavity generation damages tissue, fractures bones, and punctures the blood vessels and nerves. For instance, a stab wound posterior to the anterior axillary line would more likely impact the parenchyma but not the critical renal regions (36). Despite the known differences in the pathogenesis of blunt and penetrating trauma, we observed that the type of trauma did not affect in-hospital complications or predisposition to IRBV in our study. This calls into question the above-mentioned repercussions of differences in the injury mechanism for different types of trauma (blunt or penetrating) and their impact on IRBV, necessitating additional research into how stress is conveyed in different trauma types and mechanisms, especially given the anatomical location of the kidney and approach to vital renal sections (7, 20).

In our study, patients treated with IRBV had a higher rate of cardiac arrest. In many circumstances, cardiac arrest (CA), an underlying disease, reduced renal perfusion due to trauma, and other renal stressors may affect renal function, leading to a higher risk of iHRC (37). Even after adjusting for several other confounders, in-hospital CA was independently associated with a higher risk of iHRC. Our findings substantiate those of previous studies reporting an indisputable association between cardiovascular risk and renal failure (38, 39). However, the significance of CA in in-hospital renal complications requires further investigation in well-designed studies to draw implications for management recommendations (40, 41).

Renal injuries are known to increase the likelihood of adverse outcomes in the near term, and studies have also shown that these risks, as well as those of mortality and renal dysfunction, linger even after patients are released from the hospital (18, 33, 34, 42). Comorbidities and trauma-related factors seem to significantly affect the severity of this risk (36). In our case, half of the IRBV patients had coexisting chest injuries (AIS severity ≥3) and 25% had extremity injuries. Potential renal stressors should be avoided, and proper hemodynamic care and follow-up of patients susceptible to iHRC are necessary (42). The treatment of IRBV in the trauma setting, identification of therapeutic targets, and provision of proper follow-up all further merit investigations (42, 43).

### Strengths and limitations

This is the first NTDB study on traumatic IRBV. All types of trauma, mechanisms, and renal injuries were included. We conducted a comprehensive analysis of this population, taking into account their demographics and the circumstances of their injuries, classifying their injuries as either penetrating or blunt, and looking at mortality rates and other complications while they were hospitalized. Prospective research on appropriate therapy for IRBV and its related effects is needed in the future.

Owing to the retrospective nature of the study, we were unable to control for potential confounding factors. There are several caveats to using NTDB. Because the NTDB is not a representative sample of the population, the findings can only be applied to NTDB-participating institutions, which tend to be larger facilities with experience in treating severe traumatic cases. The data were freely available. Therefore, there is a possibility of bias owing to the periodic assessment of specific data factors, discrepancies across hospitals, and inaccurate or incomplete information. Depending on the accuracy of the data provided by individual hospitals and the extent to which individual hospitals implement national trauma-reporting guidelines, there may be gaps, errors, or incomplete information. Imaging, laboratory results, long-term follow-up information, and comorbidities are only a few examples of potentially underrepresented parts of patients' medical history in the NTDB. The high number of observations in our sample makes us vulnerable to Type I errors. Coexisting liver, spleen, and pancreatic injuries were not evaluated, and their outcomes were not compared with those of more severe coexisting injuries. Notably, we included concomitant injuries to other body regions. Decisions to exclude these other organs (such as the liver, spleen, or pancreas) helped keep the analysis focused on the research topic at hand: the relationship between IRBV and renal complications during hospitalization. Furthermore, we did not differentiate between the outcomes and methods of intervention. The surgeon's skill has a significant role in both patient results and care. The fact that we could not obtain these data also represents a limitation. Moreover, if we limited our analysis to cases with isolated kidney injury, our sample size would be much smaller, and the findings would have less validity.

## Conclusion

This study analyzed the relationship between pre-existing renal complications, IRBV, and the risk of developing iHRC. Within the NTDB, we found that 0.6% of patients had IRBV, with blunt trauma being the leading cause of injury in most cases. After controlling for ISS and other blood vessel injuries, our results showed that IRBV still affected the risk of in-hospital renal complications. IRBVs use did not increase the risk of in-hospital mortality. Specialized renal treatment and close follow-up are required for IRBV victims because of the long- and short-term effects of cardiovascular, renal, and hemodynamic issues. Additional well-designed studies are needed to derive implications for care recommendations from our study, which also underscores the impact of CA on in-hospital renal problems. Our study also emphasizes the significance of cardiac arrest in hospital-acquired renal complications, which necessitates further exploration in well-designed prospective studies to draw management recommendations.

## Data Availability

The data analyzed in this study is subject to the following licenses/restrictions: NTDB, its member hospitals, and individuals responsible for reporting and maintaining the registry data. Statements Needed for NTDB: The American College of Surgeons retains copyright ownership of the NTDB. Requests to access these datasets should be directed to n.alzerwi@mu.edu.sa.
