# Supplementary material for: Traumatic injuries to the renal blood vessels and in-hospital renal complications in patients with penetrating or blunt trauma
Source: Front Surg. 2023 May 31;10:1134945. doi: 10.3389/fsurg.2023.1134945 (PMC10264777; doi:10.3389/fsurg.2023.1134945)
Supplement: Supplementary file 1 [file Table1.docx]

# Table S1: Patient demographic and injury characteristics stratified by trauma type

| Variables |  | Penetrating(N = 119) | (N = 2) | P-value |
| --- | --- | --- | --- | --- |
| Length of stay (days) |  | 8.0 (1.0, 18.0) | 5.0 (5.0, 5.0) | 0.212 |
| Age>=65 | 56 (11.5%) | 4 (3.4%) | 0 (0.0%) | 0.026 |
| Mechanism |  |  |  | <0.001 |
| MVT | 342 (72.9%) | 0 (0.0%) | 0 (0.0%) |  |
| Firearms | 0 (0.0%) | 102 (85.7%) | 0 (0.0%) |  |
| Fall | 65 (13.9%) | 0 (0.0%) | 0 (0.0%) |  |
| Cut/Peirce | 0 (0.0%) | 17 (14.3%) | 0 (0.0%) |  |
| Others | 62 (13.2%) | 0 (0.0%) | 2 (100.0%) |  |
| Intent |  |  |  | <0.001 |
| Unintentional | 466 (96.9%) | 6 (5.0%) | 2 (100.0%) |  |
| Self-inflicted | 6 (1.2%) | 7 (5.9%) | 0 (0.0%) |  |
| Assault | 8 (1.7%) | 103 (86.6%) | 0 (0.0%) |  |
| Others | 1 (0.2%) | 3 (2.5%) | 0 (0.0%) |  |
| DUC | 89 (18.5%) | 49 (41.2%) | 1 (50.0%) | <0.001 |
| In-hospital renal complications | 30 (6.1%) | 10 (8.4%) | 0 (0.0%) | 0.627 |
| In-hospital cardiac arrest | 35 (7.3%) | 15 (12.6%) | 0 (0.0%) | 0.154 |

DUC: Died under care, MVT: Motor Vehicle Trauma
